# Effects of Psychological Intervention on Perioperative Quality of Life and Serum PSA and FPSA Levels of Patients with Prostate Cancer Treated with Integrated Traditional Chinese and Western Medicine

**DOI:** 10.1155/2021/9286905

**Published:** 2021-11-26

**Authors:** Xifeng Sun, Yi Lu, Hongxia Zhu, Rui Li, Donghua Zhang, Kunfang Pang

**Affiliations:** ^1^Department of Clinical Laboratory, Weifang People's Hospital, Weifang 261041, China; ^2^Sterilization Supply Room, Qingdao Eighth People's Hospital, Qingdao 266000, China; ^3^Department of Operation Room, Rizhao Hospital of TCM, Rizhao 276800, China; ^4^Department of Endocrinology, Zhangqiu District People's Hospital, Jinan 250200, China; ^5^Tumor-chemotherapy Department, Zhangqiu District People's Hospital, Jinan 250200, China; ^6^Department of Anesthesiology, Qingdao Hospital of Traditional Chinese Medicine, Qingdao Hiser Hospital, Qingdao 266033, China

## Abstract

**Objective:**

To observe the effects of psychological intervention on the perioperative quality of life and serum prostate-specific antigen (PSA) and free PSA (FPSA) levels in patients with prostate cancer treated with integrated traditional Chinese and Western medicine.

**Method:**

A total of 208 prostate cancer patients were selected and randomly divided into a study group with 104 cases and a control group with 104 cases. The control group received a plan of basic nursing combined with integrated traditional Chinese and Western medicine, and the study group received psychological intervention on the basis of the control group. Negative emotion, pain degree, quality of life, maximum urine flow rate, residual urine volume, International Prostate Symptom Score (IPSS), and incidence of adverse reactions were compared between the two groups before and after the treatment. The levels of PSA and FPSA and the long-term efficacy of the two groups of patients before and after treatment were compared.

**Results:**

After nursing, Hamilton Anxiety Scale (HAMA) score, Hamilton Depression Scale (HAMD) score, pain degree, maximum urine flow rate, residual urine volume, IPSS score, emotional function, social function, role function, and physical function scores of patients in two groups were decreased, and the decrease was more significant in the study group. After treatment, serum PSA and FPSA levels in the study group were obviously lower than those in the control group. The two-year cumulative survival rate of the study group was higher than that of the control group. There was no significant difference in the cognitive function score and incidence of adverse reactions between the two groups.

**Conclusion:**

Psychological intervention combined with traditional Chinese and Western medicine in the treatment of prostate cancer can effectively improve the patient's psychological state, reduce the degree of pain in patients, improve the therapeutic effect and the quality of life of patients, and significantly reduce serum PSA and FPSA levels, which could lead to a prolonged life.

## 1. Introduction

Prostate cancer is a common multiple malignant tumor in men. Its clinical manifestations include dysuria, slow urine flow, incontinence, fine urinary line, hematuria, impotence, and other symptoms, and it is highly invasive and prone to metastasis [1]. In recent years, the incidence of prostate cancer in China has shown the characteristics of increasing incidence, rapid disease progression, higher mortality, and younger, posing a severe threat to the life and health of patients [1]. Prostate cancer can be caused by many factors, such as family genetics and bad eating habits and living habits [2]. At the time of clinical diagnosis, many patients were already in the advanced stage when the disease was diagnosed because there were no clinical symptoms at the early stage, missing the best opportunity for radical surgery [3]. In addition, with the development of the disease, the tumor gradually increased, bringing some challenges to the treatment of prostate cancer [3]. Transurethral green laser vaporization of the prostate has a good effect on prostate cancer treatment, effectively relieving urethral obstruction and alleviating patients' clinical symptoms [4]. However, Lai et al. suggested that this surgical treatment still cannot completely remove the tumor tissue, and the remaining tissue can continue to grow under the stimulation of various factors, causing the urinary tract to be compressed again [5]. And patients in the perioperative period usually have anxiety, fear, and other emotions, affecting the smooth operation and postoperative recovery [6]. Thus, effective psychological nursing is crucial. Traditional Chinese medicine (TCM) is an important means of treating diseases in China. It contains rich practical experience and medical technology of ancient medical scientists, and TCM has made great progress in the treatment of prostate cancer in modern medicine [7]. Prostate cancer belongs to the category of “uroschesis,” “stranguria,” and “hematuria” in TCM, and its treatment should detoxicate and abscise mass, activate blood to remove blood stasis, and strengthen body resistance to consolidate constitution [8]. Studies have shown that the combination of traditional Chinese and Western medicine has a better therapeutic effect on the treatment of prostate cancer [9]. In this study, psychological intervention combined with integrated traditional Chinese and Western medicine was used to treat prostate cancer, aiming to observe its influence on perioperative quality of life and serum indicators of prostate cancer patients. The report is as follows.

## 2. Materials and Methods

### 2.1. Normal Information

A total of 208 prostate cancer patients admitted at Weifang People's Hospital, Weifang, China, from July 2016 to July 2018 were selected and randomly divided into a study group and a control group with 104 cases each, all of which were confirmed by pathological diagnosis. The age range in the study group was 53–77 years, with an average of 66.53 ± 8.06 years, and the course of illness was 7–28 months, with an average of 11.83 ± 4.76 months. Clinical staging: 23 cases in stage I, 48 cases in stage II, and 33 cases in stage III. There were 10 cases of squamous cell carcinoma and 94 cases of adenocarcinoma. Distant metastasis occurred in 71 cases, and no distant metastasis occurred in 33 cases. The age range in the control group was 55–76 years, the average age was 65.24 ± 8.83 years, the course of the disease was 6 to 30 months, and the average course of the disease was 11.28 ± 4.35 months. Clinical staging: 26 cases in stage I, 47 cases in stage II, and 31 cases in stage III. There were 7 cases of squamous cell carcinoma and 97 cases of adenocarcinoma. Remote metastasis occurred in 69 cases, and no distant metastasis occurred in 35 cases. There was no statistically significant difference in clinical data between the two groups of patients in terms of age, course of the disease, clinical stage, and remote metastasis, and they were comparable. This study was approved by the ethics committee of Weifang People's Hospital, Weifang, China. This study was conducted under the Declaration of Helsinki.

### 2.2. Inclusion and Exclusion Criteria

Inclusion criteria were as follows: (1) all meet the relevant diagnostic criteria of prostate cancer in the “Guidelines for the Diagnosis and Treatment of Urological Diseases in China” [10] and are confirmed by B-assisted prostate biopsy; (2) age <80 years; (3) IPSS scores are >8 points; (4) patients signed an informed consent form. Exclusion criteria were as follows: (1) patients with severe heart, lung, liver, and other organ dysfunction and neurological diseases; (2) patients with other malignant tumors; (3) patients with lymphatic system, bone marrow system, and autoimmune diseases; (4) patients allergic to the drugs used in this study.

### 2.3. Treatment Method

Patients in the control group were given basic nursing combined with integrated traditional Chinese and Western medicine treatment, and patients in the study group were treated with psychological intervention on the basis of the control group.

### 2.4. Basic Nursing

Dietary taboos information was provided and explained to patients and their family members. Clean and tidy ward conditions were provided for patients, and patients were guided to use drugs rationally.

### 2.5. Integrated Traditional Chinese and Western Medicine Treatment Methods

After the patient is admitted at the hospital, detailed understanding of his personal data, time of illness, emotional state, and family relationship should be known in detail to evaluate patients' ability of daily living and psychological state. For patients with paranoia, depression, fear, anxiety, and resistance, psychological quantitative assessment should be carried out in time, and the details should be recorded. Western medicine treatment: patients were given flutamide tablets orally, 3 times per day, 250 mg per time. TCM treatment: the main ingredients of TCM decoction include *Polyporus* 10 g, *Astragalus* 30 g, *Hedyotis diffusa* herba 15 g, *Curcuma* 10 g, *Scutellaria baicalensis* 35 g, whole scorpion 12 g, *Polygonatum* 16 g, *Achyranthes bidentata* 16 g, *Polygonum cuspidatum* 15 g, Rhizoma Atractylodis Macrocephalae 11 g, and *Poria cocos* 10 g. The aforementioned TCM was decocted to 200 ml, 1 dose/d (in the morning and evening, respectively), and the course of treatment was 3 months.

### 2.6. Psychological Intervention Treatment Methods

(1) Preoperative nursing: the nurse actively communicates with the patient and explains the disease situation, treatment methods, the operation principle, anesthesia methods, and precautions to the patient and family members, so as to eliminate the patient's worries, reduce the patient's nervousness, and enable the patient to receive treatment in a good state of mind. In the evening, one day before the operation, the patient was told to eat easy-to-digest food for dinner, fast for 12 hours, and water restriction for 4 hours before the operation, allowing the patient to rest as soon as possible to ensure sleep. For some patients with poor sleep, appropriate sedative and hypnotic drugs can be used. (2) Postoperative nursing: the nurse guides the family members or the caregiver to provide a nutritious and reasonable diet and assists the patient with moderate exercise. The nurse patiently urges the patient to strictly follow the doctor's advice and take medicine on time. When providing nursing services to patients, they should be active, enthusiastic, sincere, and patient and enhance patients' compliance with treatment. A quiet, comfortable, and safe treatment environment was provided to patients. Family members, relatives, and friends of patients should be educated on hygiene and psychology, are required to understand prostate cancer and the patient's condition, not irritate or demand the patient, and provide financial support to make the patient feel the warmth of the family. Regular lectures are held to explain some successful cases of treatment and articles that inspire the soul to enhance the confidence and determination of patients to overcome the disease. (3) Continuing nursing: after the patient is discharged from the hospital, a monthly follow-up visit should be conducted to understand the patient's medication and recovery status. If there are adverse reactions and poor medication compliance, correct guidance should be given to help the patient build confidence in treatment. The evaluation, care, treatment, follow-up, and other information of each patient were organized and archived.

### 2.7. Observation Index

(1) The negative emotional state of the two groups of patients before and after nursing was compared. The evaluation is based on the HAMA and HAMD scales [11], with a total score of 0 to 56 points. The lower the score, the milder the patient's anxiety and depression. (2) The pain degree and quality of life of the two groups of patients before and after nursing were compared. The visual analogue scoring (VAS) method was used to evaluate the pain degree of the two groups [12]. The score ranged from 1 to 10 points. The higher the score, the more obvious the pain degree. The European QLQ-C30 functional scale was used to evaluate the recovery of the two groups of patients from the five dimensions of emotional function, cognitive function, social function, role function, and physical function [13]. Each item was scored 100 points. The higher the score, the better the corresponding functional recovery. (3) The PSA and FPSA indexes of the two groups before and after treatment were compared. In the morning, 5 mL of venous blood from the patient's elbow was collected on an empty stomach, centrifuged at 3500 r/min for 15 minutes, and the upper serum was taken and stored in a low-temperature refrigerator at −60°C for testing. Enzyme-linked immunoassay (ELISA) was used to detect the serum PSA and FPSA contents of patients. The kit was purchased from the American Biotech Reagent Co., Ltd. The operation steps were strictly in accordance with the instructions. (4) The maximum urine flow rate, residual urine volume, and prostate symptom scores (International Prostate Symptom Score, IPSS) were compared between the two groups [14]. The maximum urine flow rate and residual urine volume of the two groups were counted before and after nursing, and the IPSS was used for evaluation. The score was proportional to the severity of prostate symptoms: 0–7 points: mild; 8–19 points: moderate; 20–35 points: severe. (5) The long-term efficacy of the two groups of patients was compared. Follow-up for two years, once a month, and the two-year cumulative survival rate of the two groups were recorded and calculated. (6) The incidence of adverse reactions (including gastrointestinal reactions, liver damage, breast development, infections, and bladder spasm) between the two groups was compared.

### 2.8. Statistical Analysis

SPSS 22.0 software performs statistical analysis on the data. Measurement data were expressed as mean ± standard deviation ($\bar{x}\pm s$), and comparison between groups was performed by paired sample *t*-test. The enumeration data were expressed in terms of the number of cases and the rate (%), and the *χ*^2^ test was used for comparison between groups. The difference is statistically significant with *p* < 0.05.

## 3. Results

### 3.1. Comparison of Negative Emotions between Two Groups of Patients before and after Nursing

**Figure 1:**
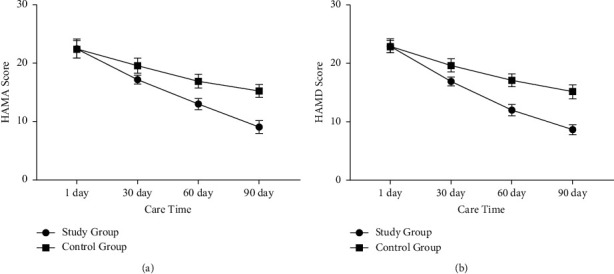
Comparison of negative emotions between the two groups of patients before and after nursing. (a) The comparison of HAMA scores between the two groups of patients before and after nursing. (b) The comparison of HAMD scores between the two groups of patients before and after nursing.

After one day of nursing, there was no significant difference in the HAMA score and HAMD score between the two groups. After 30 days, 60 days, and 90 days of nursing, the HAMA score and HAMD score of the two groups decreased, and the scores of patients in the study group decreased more significantly than the control group (*p* < 0.05) (Figure 1).

### 3.2. Comparison of Pain Degree and Quality of Life between Two Groups before and after Nursing

Before nursing, there was no significant difference in pain degree and the scores of the quality of life between the two groups. After 30 days, 60 days, and 90 days of nursing, the pain degree of the two groups decreased, and the decrease in the study group was more significant than that in the control group (*P* < 0.05) (Table 1). After nursing, there was no statistically significant difference in cognitive function scores between the two groups, and there was a decline in emotional function, social function, role function, and physical function scores, and the decline in the study group was even more significant (*P* < 0.05) (Table 2).

### 3.3. Serum PSA and FPSA Levels Were Compared between the Two Groups

**Figure 2:**
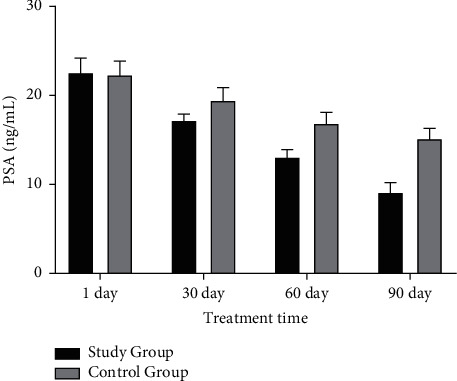
Comparison of PSA levels between the two groups before and after treatment.

**Figure 3:**
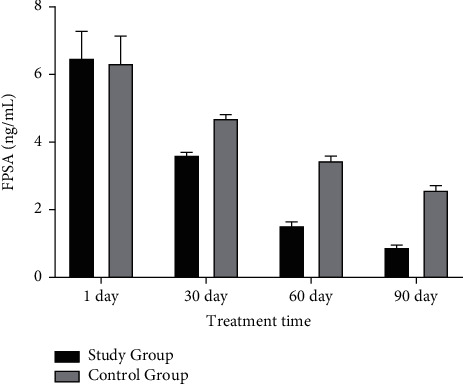
Comparison of FPSA levels between the two groups before and after treatment.

After 1 day of treatment, there was no significant difference in serum PSA and FPSA levels between two groups (Figures 2 and 3). With the increase of treatment time, the levels of the two indexes decreased (Figures 2 and 3). After 90 days of treatment, serum PSA and FPSA levels in the study group were obviously decreased versus the control group (Figures 2 and 3).

### 3.4. Comparison of Maximum Urine Flow Rate, Residual Urine Volume, and IPSS Score between Two Groups before and after Nursing

Before nursing, there were no significant differences in maximum urine flow rate, residual urine volume, and IPSS score between two groups (Table 3). After nursing, each index was decreased, and the decrease in the study group was more obvious than the control group (Table 3).

### 3.5. Comparison of the Incidence of Adverse Reactions between the Two Groups

In the study group, 23 patients had gastrointestinal reactions, 21 had liver damage, 17 had breast development, 6 had an infection, and 2 had bladder spasm (Table 4). In the control group, there were 27 cases of gastrointestinal reactions, 24 cases of liver damage, 23 cases of breast development, 10 cases of infection, and 7 cases of bladder spasm (Table 4). There was no statistically significant difference in the incidence of adverse reactions between the two groups of patients in gastrointestinal reactions, liver damage, breast development, infection, and bladder spasm (Table 4).

### 3.6. Comparison of the Long-Term Efficacy of the Two Groups

**Figure 4:**
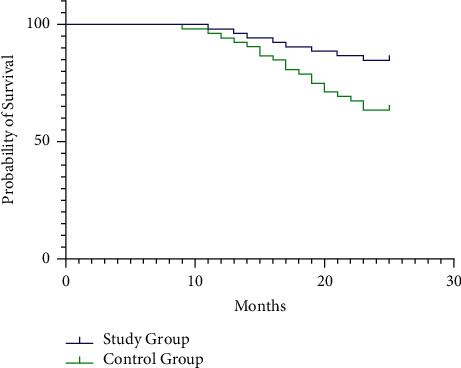
Comparison of two-year cumulative survival rates between the two groups.

The two-year cumulative survival rate in the study group (84.62%) was higher than that in the control group (63.46%) (Figure 4).

## 4. Discussion

The clinical pathogenesis of prostate cancer is unclear and related to many factors [15]. Prostate cancer patients usually have anxiety, fear, depression, and other negative emotions, as well as the reduction of the androgen level after surgical treatment, affecting sexual function, which will not only cause inferiority but also affect the recovery of the body. Hence, the quality of psychological care has higher requirements [16, 17]. This study showed that, after nursing, the HAMA and HAMD scores of the study group were significantly lower than those of the control group, suggesting that psychological care can improve patients' negative emotions. In this study, the mental state of patients was evaluated before nursing, which avoided the blindness of nursing and improved the pertinence and applicability of nursing measures. Here, the pain degree of patients in the two groups was obviously reduced, and the quality of life was notably improved, and the improvement of the study group was more significant, indicating that the actual situation of patients should be fully understood in psychological nursing to prevent their mood deterioration. Some patients have psychological resistance to treatment, worry about physical disability after surgery, and inferiority mentality, so patients' wrong understanding should be corrected to enhance their confidence in life during psychological nursing. Their psychological state should be closely monitored in nursing [18]. Compared with general nursing, psychological intervention in this study improves the purpose of nursing, avoids the waste or shortage of nursing resources, and fully meets the needs of patients.

In recent years, clinicians have gradually begun to pay attention to the use of Chinese medicine as the adjuvant treatment in prostate cancer. According to TCM approaches, prostate cancer is mainly caused by the weakness of the human body to protect the body surface, as well as external evil, wind, cold, wet, heat, and other internal invasions caused by deficiency [19]. Treatment should follow the principles of supporting righteous qi and regulating the body's disease resistance, fighting poison with poison to dissipate agglomeration, promoting blood circulation, and dissipating blood stasis [20]. In this study, *Polyporus* eliminated dampness and diuresis, facilitated urination, and dissolved dampness for subsiding swelling; *Astragalus* can not only replenish qi of the whole body but also good tonify qi of the muscle surface; *Hedyotis diffusa* herba can clear heat and remove toxicity, diuresis, and dehumidification; *Curcuma* promotes blood circulation for removing blood stasis; *Scutellaria baicalensis* can clear away heat and detoxify and treat diseases caused by dampness and heat accumulation in the body; whole scorpion can treat convulsions; also, it has analgesic effects; *Polygonatum* can supplement qi and nourish yin, strengthen the spleen, moisten the lung, and reinforce the kidney; *Achyranthes bidentata* promotes blood circulation for removing blood stasis, reinforces the liver and kidney, strengthens muscles and bones, and induces diuresis for treating stranguria; *Polygonum cuspidatum* removes dampness, clears heat and detoxification, dispels stasis, stanches pain, and resolves phlegm for relieving cough; Rhizoma Atractylodis Macrocephalae could invigorate qi, strengthen the spleen, remove moisture from the body and stop sweating; *Poria cocos* eliminates dampness and diuresis, tonifies the spleen, harmonizes the stomach, and makes peace of mind [21–26]. The combination of multiple drugs can improve the immune function of the patient's body, thereby alleviating the patient's clinical symptoms. After treatment, the serum PSA and FPSA levels of the two groups were decreased significantly compared to before treatment. Serum PSA is a specific marker of prostate cancer. It is a serine protease produced in prostate epithelial cells, and its normal function is to help hydrolyze and liquefy semen clots [27]. PSA is associated with male fertility, and the serum PSA level of prostate disease will increase [28]. It shows that the combination of traditional Chinese and Western medicine combined with psychological intervention has significant therapeutic effects in patients with prostate cancer, significantly improving the patients' serum PSA and FPSA levels and improving the body's immunity. After nursing, the maximum urine flow rate, residual urine volume, and IPSS score of the two groups of patients decreased, and the decrease in the study group was more predominant than that of the control group. It further shows that the combination of psychological intervention and integrated traditional Chinese and Western medicine in this study has good effects on prostate cancer, and it is worthy of clinical application. There was no significant difference in the occurrence of adverse reactions between the two groups after treatment, indicating that the safety of the two treatment methods was basically the same.

## 5. Conclusion

In summary, the combination of psychological intervention and integrated traditional Chinese and Western medicine in the treatment of prostate cancer can significantly improve the therapeutic effects, regulate the body's immune function, improve the quality of life of patients, and reduce the side effects of Western medicine.

## Figures and Tables

**Table 1:** Comparison of pain degree between the two groups of patients before and after nursing ($\bar{x}\pm s$).

Group	Cases	Before nursing	Nursing for 30 days	Nursing for 60 days	Nursing for 90 days
Study	104	8.03 ± 1.14	5.74 ± 0.86	3.26 ± 0.69	1.85 ± 0.56
Control	104	8.17 ± 1.08	6.78 ± 0.94	5.52 ± 0.88	4.81 ± 0.93
*t*		0.026	4.957	7.660	12.381
*P*		0.743	0.001	<0.001	<0.001

**Table 2:** Comparison of the quality of life of the two groups of patients before and after care ($\bar{x}\pm s$).

Project	Time	Study	Control	*t*	*P*
Emotional function	Before nursing	52.37 ± 7.65	53.14 ± 7.87	0.324	>0.05
	After nursing	78.54 ± 8.53^*∗*^^△^	61.94 ± 7.15^*∗*^	10.374	<0.05

Cognitive function	Before nursing	63.54 ± 6.63	65.42 ± 6.26	0.463	>0.05
	After nursing	66.87 ± 6.91	67.25 ± 7.12	0.527	>0.05

Social function	Before nursing	55.68 ± 5.62	54.34 ± 5.73	0.297	>0.05
	After nursing	78.56 ± 7.88^*∗*^^△^	66.48 ± 6.54^*∗*^	7.872	<0.05

Role function	Before nursing	53.43 ± 6.71	54.29 ± 6.82	0.782	>0.05
	After nursing	73.62 ± 7.53^*∗*^^△^	62.85 ± 6.31^*∗*^	4.965	<0.05

Physical function	Before nursing	51.03 ± 5.58	51.27 ± 5.63	0.164	>0.05
	After nursing	68.65 ± 6.34^*∗*^^△^	59.56 ± 6.17^*∗*^	7.313	<0.05

Compared with the same group before treatment, ^*∗*^*P* < 0.05; compared with the control group, Δ*P* < 0.05.

**Table 3:** Comparison of maximum urine flow rate, residual urine volume, and IPSS score between the two groups before and after nursing ($\bar{x}\pm s$).

Indexes	Study	Control	*t*	*P*
*Maximum urine flow rate (ml/s)*				
Before nursing	9.54 ± 3.67	9.71 ± 3.59	0.234	>0.05
After nursing	20.13 ± 3.34	13.46 ± 3.28	10.315	<0.05

*Residual urine volume (ml)*				
Before nursing	47.63 ± 7.89	48.15 ± 7.93	0.762	>0.05
After nursing	13.87 ± 3.36	27.61 ± 4.74	12.370	<0.05

*IPSS score*				
Before nursing	22.64 ± 2.98	22.37 ± 2.65	0.523	>0.05
After nursing	5.16 ± 0.45	9.68 ± 1.06	7.662	<0.05

**Table 4:** Comparison of the incidence of complications between the two groups (*n*).

Group	Cases	Gastrointestinal reactions	Liver damage	Breast development	Infection	Bladder spasm
Study	104	23	21	17	6	2
Control	104	27	24	23	10	7
*χ* ^2^		0.421	0.255	1.114	1.083	2.903
*P*		0.516	0.613	0.291	0.298	0.088

## Data Availability

The datasets used and/or analyzed during the present study are available from the corresponding author upon reasonable request.

## References

[1] Grozescu T., Popa F. (2018). Prostate cancer between prognosis and adequate/proper therapy. *Journal of Medicine & Life*.

[2] Sehn J. K. (2018). Prostate cancer pathology: recent updates and controversies. *Missouri Medicine*.

[3] Daniyal M., Siddiqui Z. A., Akram M., Asif H. M., Khan A. (2018). Epidemiology, etiology, diagnosis and treatment of prostate cancer. *Asian Pacific Journal of Cancer Prevention Apjcp*.

[4] Brassetti A., Nunzio C. D., Delongchamps N. B., Fiori C., Tubaro A. (2020). Green light vaporization of the prostate (PVP): is it an adult technique?. *The Italian Journal of Urology & Nephrology*.

[5] Lai S., Peng P., Diao T. (2020). Comparison of photoselective green light laser vaporisation versus traditional transurethral resection for benign prostate hyperplasia: an updated systematic review and meta-analysis of randomised controlled trials and prospective studies. *BMJ Open*.

[6] Cheah W. L., Ling N. C., Chang K. H. (2020). The supportive care needs for prostate cancer patients in sarawak. *Chinese Journal of Clinical Oncology*.

[7] Lin Y. H., Chen K. K., Chiu J. H. (2020). Trends in Chinese medicine use among prostate cancer patients under national health insurance in Taiwan: 1996-2008. *Integrative Cancer Therapies*.

[8] Lin Y. H., Chen K. K., Chiu J. H. (2020). Prevalence, patterns, and costs of Chinese medicine use among prostate cancer patients: a population-based study in Taiwan. *Integrative Cancer Therapies*.

[9] Jin Y. (2019). Current opinion and mechanistic interpretation of combination therapy for castration-resistant prostate cancer. *Asian Journal of Andrology*.

[10] Meo A. D., Pasic M., Yousef G. M. (2019). Proteomics and peptidomics: moving toward precision medicine in urological malignancies. *Oncotarget*.

[11] Nissen E. R., O’Connor M., Kaldo V., Hjris I., Mehlsen M. (2019). IInternet-delivered mindfulness-based cognitive therapy for anxiety and depression in cancer survivors: a randomized controlled trial. *Psycho-Oncology*.

[12] Charalambous A., Giannakopoulou M., Bozas E., Paikousis L. (2019). Parallel and serial mediation analysis between pain, anxiety, depression, fatigue and nausea, vomiting and retching within a randomised controlled trial in patients with breast and prostate cancer. *BMJ Open*.

[13] Guan T., Santacroce S. J., Chen D., Song L. (2019). Illness uncertainty, coping, and quality of life among patients with prostate cancer. *Psycho-Oncology*.

[14] Tomita N., Oze I., Hidetoshi S. H., Tachibana H., Hayashi N. (2020). International prostate symptom score (IPSS) change and changing factor in intensity-modulated radiotherapy combined with androgen deprivation therapy for prostate cancer. *Nagoya Journal of Medical Science*.

[15] Rebbeck T. R. (2018). Prostate cancer genetics: variation by race, ethnicity, and geography. *Seminars in Radiation Oncology*.

[16] Schouten B., Avau B., Bekkering G. E., Vankrunkelsven P., Hecke A. V. (2018). Systematic screening and assessment of psychosocial well-being and care needs of people with cancer. *Cochrane Database of Systematic Reviews*.

[17] Cuevas A. G., Trudel-Fitzgerald C., Cofie L., Zaitsu M., Allen J., Williams D. R. (2018). Placing prostate cancer disparities within a psychosocial context: challenges and opportunities for future research. *Cancer Causes & Control*.

[18] Frankland J., Brodie H., Cooke D., Foster C., Richardson A. (2018). Follow-up care after treatment for prostate cancer: evaluation of a supported self-management and remote surveillance programme. *BMC Cancer*.

[19] Huijuan C., Yujie M., Xun Li, Wang S. (2018). A systematic review of randomized controlled trials on oral Chinese herbal medicine for prostate cancer. *Plos One*.

[20] Gong X., Wang J.-S, Yu X.-D (2018). Assessment of the efficacy of Chinese patent medicine on treating pain caused by prostate cancer: a protocol for systematic review and meta analysis. *Medicine*.

[21] Lin P.-H., Lin S.-K., Hsu R.-J. (2018). Spirit-quieting traditional Chinese medicine may improve survival in prostate cancer patients with depression. *Journal of Clinical Medicine*.

[22] Wang N., Xu L., Wang J.-S. (2019). Traditional Chinese medicine on treating pain caused by prostate cancer: a systematic review and meta-analysis. *Medicine*.

[23] Wang X., Fang G., Pang Y. (2019). Chinese medicines in the treatment of prostate cancer: from formulas to extracts and compounds. *Nutrients*.

[24] Liu J.-M., Lin P.-H., Hsu R.-J. (2019). Complementary traditional Chinese medicine therapy improves survival in patients with metastatic prostate cancer. *Medicine*.

[25] Guo S., Ma B., Jiang X., Li X., Jia Y. (2019). Astragalus polysaccharides inhibits tumorigenesis and lipid metabolism through miR-138-5p/SIRT1/SREBP1 pathway in prostate cancer. *Frontiers in Pharmacology*.

[26] Bonham M. (2019). Characterization of chemical constituents in Scutellaria baicalensis with antiandrogenic and growth-inhibitory activities toward prostate carcinoma. *Clinical Cancer Research An Official Journal of the American Association for Cancer Research*.

[27] Saini S.. (2019). PSA and beyond: alternative prostate cancer biomarkers. *Cellular Oncology*.

[28] Venderbos L. D., Roobol M. J. (2019). PSA-based prostate cancer screening: the role of active surveillance and informed and shared decision making. *Asian Journal of Andrology*.

